# Study of the Chemotactic Response of Multicellular Spheroids in a Microfluidic Device

**DOI:** 10.1371/journal.pone.0139515

**Published:** 2015-10-07

**Authors:** Jose M. Ayuso, Haneen A. Basheer, Rosa Monge, Pablo Sánchez-Álvarez, Manuel Doblaré, Steven D. Shnyder, Victoria Vinader, Kamyar Afarinkia, Luis J. Fernández, Ignacio Ochoa

**Affiliations:** 1 Group of Structural Mechanics and Materials Modeling (GEMM), Centro Investigacion Biomedica en Red. Bioingenieria, biomateriales y nanomedicina (CIBER-BBN), Zaragoza, Aragon, Spain; 2 Aragón Institute of Engineering Research (I3A), University of Zaragoza, Zaragoza, Aragon, Spain; 3 Aragon Institute of Biomedical Research, Instituto de Salud Carlos III, Madrid, Madrid, Spain; 4 Institute of Cancer Therapeutics, Faculty of Life Sciences, University of Bradford, Bradford, West Yorkshire, United Kingdom; University of Illinois at Urbana-Champaign, UNITED STATES

## Abstract

We report the first application of a microfluidic device to observe chemotactic migration in multicellular spheroids. A microfluidic device was designed comprising a central microchamber and two lateral channels through which reagents can be introduced. Multicellular spheroids were embedded in collagen and introduced to the microchamber. A gradient of fetal bovine serum (FBS) was established across the central chamber by addition of growth media containing serum into one of the lateral channels. We observe that spheroids of oral squamous carcinoma cells OSC–19 invade collectively in the direction of the gradient of FBS. This invasion is more directional and aggressive than that observed for individual cells in the same experimental setup. In contrast to spheroids of OSC–19, U87-MG multicellular spheroids migrate as individual cells. A study of the exposure of spheroids to the chemoattractant shows that the rate of diffusion into the spheroid is slow and thus, the chemoattractant wave engulfs the spheroid before diffusing through it.

## Introduction

*Chemotaxis* is the process by which cells migrate along a concentration gradient towards a chemoattractant. Chemotaxis plays a critical role in many pathologies, including inflammation [1–3] and autoimmune diseases [4] as well as cancer [4–7], and also many developmental and tissue remodeling processes, including embryogenesis and wound healing [8, 9]. Therefore, techniques that enable detailed scrutiny of the chemotactic process are important tools for drug discovery as well as basic biology.

A number of different protocols are used to study cell migration and chemotaxis. The Boyden chamber assay [10] has been one of the most popular assays to study chemotactic response for many years. Although this method is versatile, it has some limitations; for example, it does not allow direct observation of cells during migration processes. A number of other techniques have been developed to monitor cell movement in chemotactic experiments, including the under-agarose gel [11], agarose spot [12], Zigmond chamber [13], Dunn chamber [14], and Insall chamber [15] assays. Although each of these assays has its own merits, they generally do not allow gradient monitoring over time, and in addition, gradient control and reproducibility can be challenging. Many of these shortcomings can be overcome by using microfluidic systems that have come to the fore as powerful tools to study chemotaxis [16–18]. A large variety of microdevices have been designed to generate chemotactic gradients, and one of the most well-known is the Premixer Gradient Generator[19]. These microdevices rely on microchannels that split and recombine two different liquids several times in order to generate the desired gradient. Other common designs to generate molecular gradients are based on the use of microjets. In such microdevices molecules are transported through small microchannels by convection to the culture area. This culture area is much larger than the microchannels, causing the transported molecules to lose momentum. Therefore, within the culture area, diffusion dominates over convection, leading to gradient generation[20]. The microdevice presented herein belongs to another type of gradient generators based on porous hydrogel membranes. Hydrogels possess a porous structure which opposes high resistance to liquid flow. However, molecules can diffuse passively through these pores, enabling the gradient generation. Microdevices designed with a central chamber to accommodate a hydrogel, and flanked by lateral microchannels to perfuse different media, have proved to be robust tools to generate gradients and study cell responses [21–26].

Whilst observations of single cell migration with the existing techniques are very useful, they are in some way distanced from more realistic cell migration scenarios, in which cells are part of a multicellular system. Many reports have shown that solid tumor cells can migrate and invade by different mechanisms than individual cells [27, 28]. For example, gliomas are examples of isolated invading tumors, whereas epithelial tumors seem to invade by collective movements [27, 29]. With these differences in mind, it is important to consider that to date; none of the previously described assay protocols allow the study of this collective cancer cell migration in multicellular spheroids in response to chemotactic gradients, which leaves a major gap in our understanding of the process. Here we present a microfluidic device of simple design that permits real time observation of multicellular spheroids embedded in a hydrogel. Moreover, it allows the study of their chemotactic behavior under conditions generated by introduction of a chemoattractant gradient through lateral microchannels. We directly compare the migration of multicellular spheroids embedded in collagen in a microfluidic system to those in a well-plate. We will show, that in response to chemotactic gradient, epithelial squamous cancer cells behave differently when cultured as isolated cells than as multicellular spheroids. This is the first example of such an investigation utilizing a microfluidic device.

## Materials and Methods

### Microdevice design and fabrication

In order to easily accommodate multicellular spheroids, microdevices were designed with 400 μm diameter inlets and with 400μm wide and 300μm high microchannels. The central microchamber is 1000μm wide and 300μm high. Microdevices were fabricated using SU–8 photolithography combined with an SU–8 to SU–8 bonding process[30]. This fabrication process was inspired by previously reported work [31–33] and is described in detail in the supporting information (S1 File). Briefly, different SU–8 layers were patterned with the desired geometry in order to create the bottom, middle and top layers. The central microchamber is separated from lateral microchannels by a series of pillars, which allow hydrogel confinement due to the hydrogel’s surface tension. When it is injected within the microdevice, the hydrogel interface gets pinned between these pillars, filling only the central microchamber without entering the lateral microchannels. After hydrogel polymerization, different media can be injected through lateral microchannels with no mixing except for passive diffusion, which allows the establishment of diffusion-controlled gradients.

### Cells and reagents

Human oral squamous cancer cell line OSC–19 [34] was a generous gift from Dr Faye Johnson, MD Anderson Cancer Center, Houston, USA. Human glioblastoma cell line U–87 MG was purchased from American Type Culture Collection (ATCC; LGC Standards, Middlesex, UK). Prior to use, both cell lines were maintained as monolayers in RPMI 1640 growth media (R7388) supplemented with 10% (v/v) fetal bovine serum (FBS)(F1051), 1 mM sodium pyruvate (S8636) and 2 mM L-glutamine (G7513) (All from Sigma–Aldrich, Dorset, UK). For the remainder of the manuscript, RPMI 1640 growth media supplemented with FBS is referred to as “complete” whereas RPMI 1640 growth media not supplemented with FBS is referred to as “incomplete”. When required, cells were trypsinized and used as a suspension in growth media.

Fluorescein diacetate (FDA) (Sigma F7378), Rhodamine B (Sigma, R6626) propidium iodide (PI) (Sigma P4170), Cascade Blue®-10 kDa and Tetramethylrhodamine (TRITC)-70 kDa dextrans (Life Technologies, D1976 and D1818) were used as solutions in phosphate-buffered saline (PBS) (Lonza BE17-516F). PBS 5X was prepared by dissolving of 1 pack of PBS powder (Sigma P3813) in 200 ml deionised water. For immunofluorescence primary anti-Ki–67 antibody (abcam ab92742), secondary donkey anti-rabbit alexa-546-conjugated (life technologies A10040) and DAPI (life technologies D1306) were used.

### Spheroid generation

OSC–19 and U–87 MG multicellular spheroids were generated using the hanging drop method [35] with methylcellulose [36]. Briefly, 12 g of high viscosity methylcellulose (Sigma, M0512) were dissolved in one liter of RPMI1640, and centrifuged at 4000 g for 2.5 hours. Only the clear supernatant was used in the next step. Cell suspension (10^5^ cells/ml) and this methylcellulose solution were mixed in a 4:1 v/v ratio, and 25 μl droplets (2x10^3^ cells) were placed on the lid of Petri dishes. Sterile water was placed on the bottom of the dishes to prevent evaporation from droplets, the lids were replaced and the Petri dishes incubated at 37°C and 5% CO_2_. After 1 day, a single well-defined spheroid was generated per drop. To ensure the response of the spheroids only depends on exposure to the external chemotactic gradient of FBS, traces of serum were removed from the spheroids prior to the experiments by washing with incomplete RPMI. To do this, each spheroid was transferred to a single well from a flat bottom 96 well-plate with 200 μl of incomplete media and allowed to stand for 30 minutes. The mean spheroid diameters were 248±18 μm for OSC–19 and 236±5 μm for U–87 MG.

### Preparation of the microdevice and well-plates

15.4 μl of collagen type I (4.88 mg/ml, Corning 354236), 0.37 μl of aqueous NaOH (1N, Sigma 655104) and 4 μl of PBS 5X were mixed and added to a suspension of fifty spheroids in 30 μl of incomplete media. The final concentration of the collagen in this stock mixture was 1.5 mg/ml. The spheroid suspension in collagen was injected into the microdevice, and 5 μl of spheroid suspension was placed on top of the inlet to prevent hydrogel evaporation during polymerization. The microfluidic device was then placed into an incubator (37°C, 5% CO_2_) for 15 minutes to allow collagen polymerization. For embedding of individual cells, a similar procedure to the one described above was used to obtain a hydrogel mixture; in this case a cell suspension of 2x10^3^ cells/μl was mixed with the collagen mixture and injected into the microdevice.

### Gradient visualization

FDA (5 μg/ml), Rhodamine B (1 μg/ml), PI (4 μg/ml), 10 kDa dextran (10 μM) or 70 kDa dextran (10 μM) were pipetted into one of the lateral microchannels whilst PBS was perfused through the other one. To visualize gradient evolution, time-lapse confocal microscopy images were taken.

### Chemotaxis experiments

**Fig 1:**
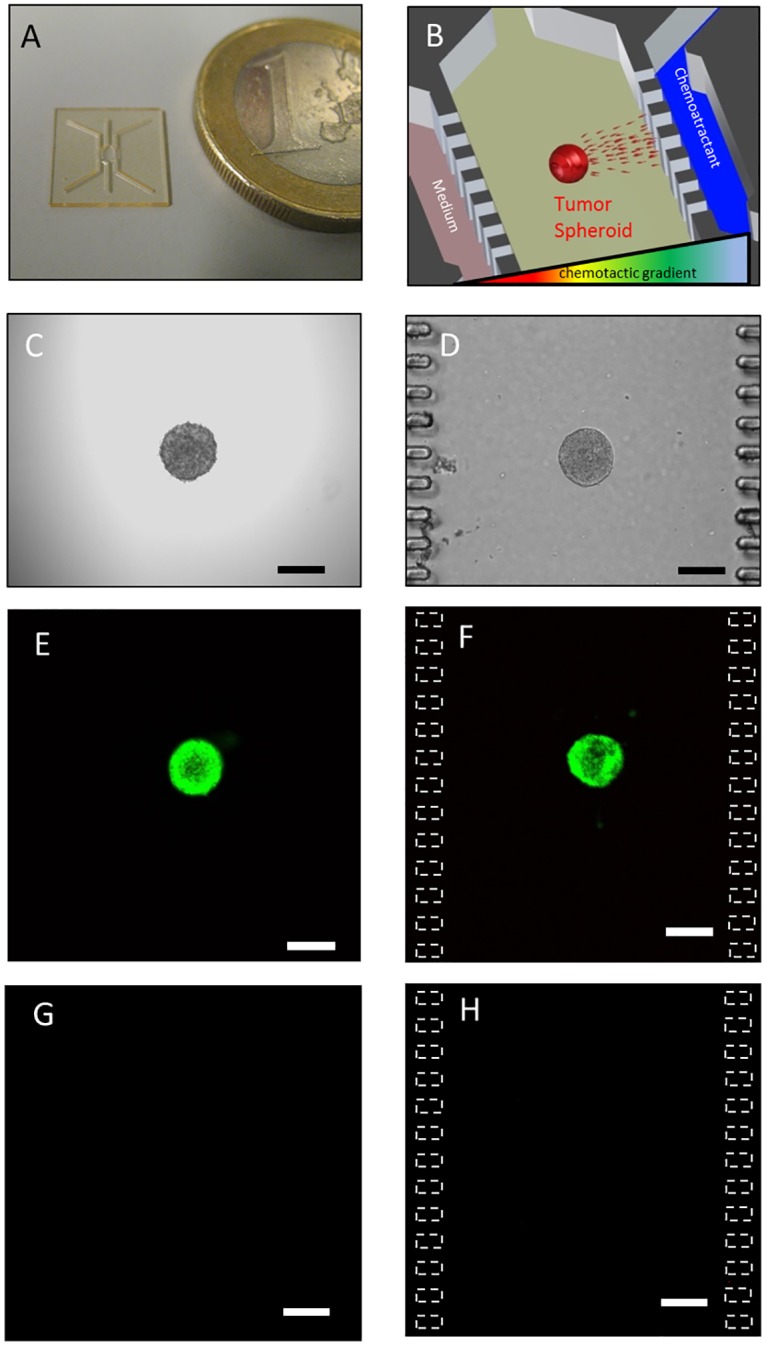
Experimental set-up. (A) SU–8 fabricated microdevice. (B) Experimental scheme. (C) OSC–19 spheroid of 2000 cells in the hanging drop. (D) Same spheroid after embedding in 1.5 mg/ml collagen hydrogel within the microdevice. Treatment of the spheroids with FDA/PI after 48 hours in a hydrogel in a 96-well plate or within the microdevice shows the spheroid is intensely green (E and F respectively) whereas less than five red cells where observed (G and H respectively), Z projection of the whole spheroid is shown. Scale bar is 200 μm.

Chemotaxis experiments were performed within the SU–8 based microdevices (Fig 1A). Complete growth media was perfused through one lateral microchannel, whereas incomplete growth media was perfused through the opposite side (Fig 1B). Microdevices were observed on the microscope with temperature and CO_2_ control systems, with conditions set at 37°C and 5% CO_2_. Brightfield images were taken every 5 minutes.

### Immunofluorescence

Samples were fixed for 30 min with 4% paraformaldehyde (VWR J61899-AP), permeabilized using 0.1% Triton-X–100 (Sigma T8787) and blocked with 5% BSA (Sigma A2058) in PBS. Samples were then incubated overnight with primary antibody (1/50 in 2.5% BSA and 0.05% Triton X–100), and secondary antibody (1/200) was used under the same conditions. DAPI staining was performed overnight and after a washing step samples were visualized.

### Imaging and analysis

To assess cell viability and visualize the gradient, confocal images were taken using a Nikon Eclipse Ti microscope equipped with a C1 modular confocal microscope system. Images were collected at different focal planes (300 μm in the “z” direction with 10 μm steps) for each microdevice and well-plate control. In order to analyze cell viability a “Z projection” was performed using the Fiji® (http://fiji.sc/Fiji) maximum intensity projection algorithm. Isolated cell migration was tracked and quantified using the Fiji® manual tracking plugin, whereas OSC–19 spheroid migration was quantified by measuring manually the spheroid-occupied area at different times. This spheroid-occupied area was drawn to show the collective invasion under the different chemotactic conditions.

### Statistical analysis

Experiments were repeated at least three times. OSC–19 invasion results are presented as mean ± standard error. Statistical analysis was performed using SPSS software, and statistical significance was set at *p* < 0.05. The normal distribution was tested by the Kolmogorov-Smirnov test, and the Student´s t-test was used for the data analysis.

## Results and Discussion

### Spheroids are viable inside the microfluidic device

We compared the viability of cells within the spheroids inside the microfluidic device to those in a well-plate plate 24 hours after implantation by treating both with FDA/PI solutions to detect viable and dead cells. We observed that spheroids within the microdevice remained intact during injection within the microdevice. Cell viability was measured in spheroids located within the microdevice as well as in those placed in the well-plate. Results showed more than 95% of the cells were viable in both conditions (Fig 1) and no necrotic core was observed.

### Visualization of the chemo-gradient

**Fig 2:**
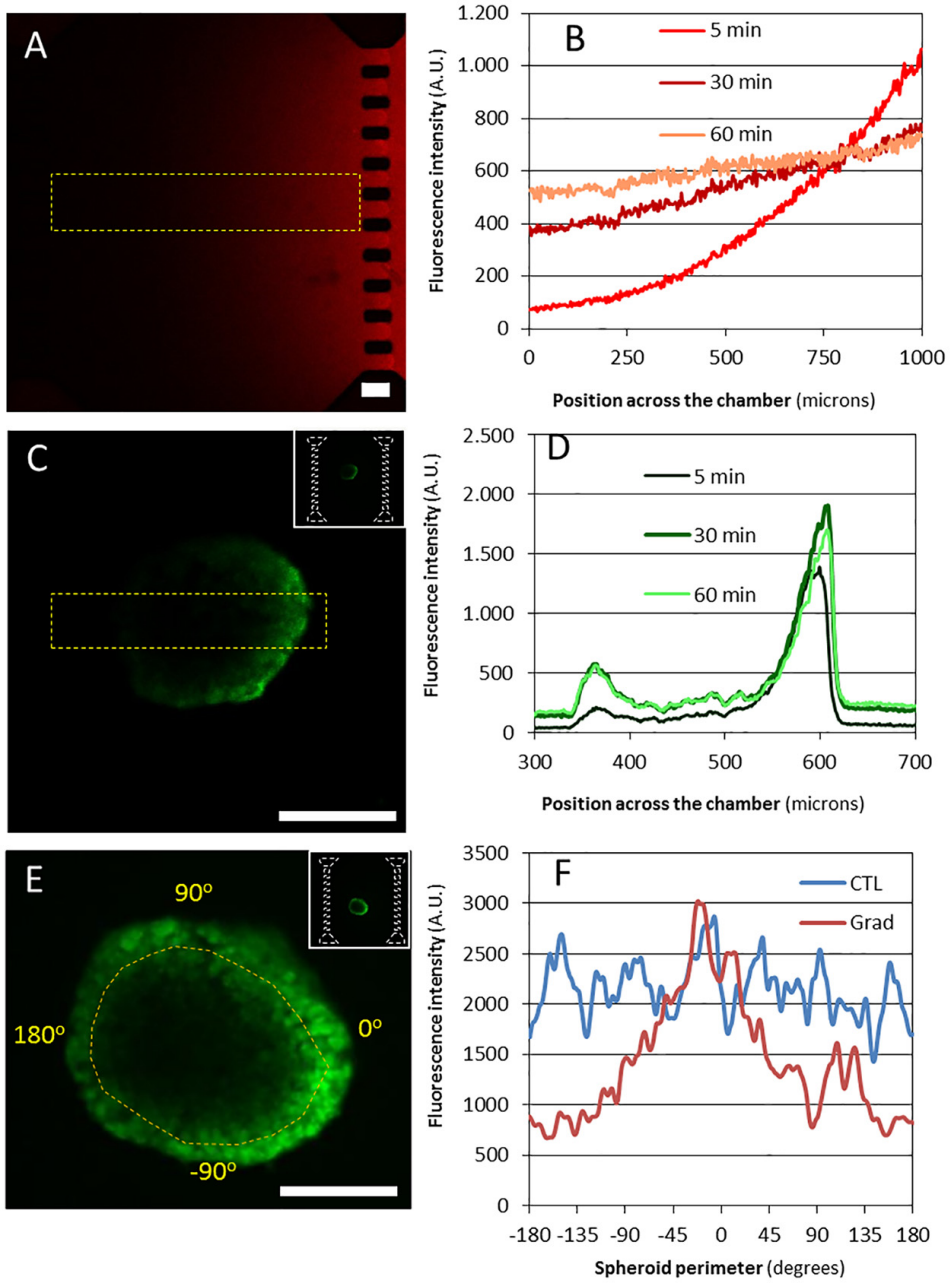
Chemical gradient across the microchamber. (A) A complete image of the microchamber 5 minutes after red fluorescent Rhodamine B was perfused through the right lateral microchannel. (B) Evolution of fluorescence intensity of Rhodamine B along the whole microchamber width (as highlighted in yellow in A) over time. (C) An image of an OSC–19 spheroid after 5 minutes of FDA perfusion through the right lateral microchannel. (D) Fluorescence intensity along the section of the spheroid (as highlighted in yellow in B) after 5 minutes of FDA perfusion. (E) Fluorescence intensity within the spheroid after 1 hour of FDA perfusion. (F) Fluorescence intensity along the spheroid perimeter after 1 hour of FDA perfusion through only one lateral microchannel (gradient condition-blue line) or through the hydrogel (control condition-red line). Images corresponding with the spheroid middle focal plane are shown. Scale bar is 100 μm.

We next set out to gauge the exposure of the spheroids and individual cells within the microdevice to a chemical gradient. To assess how this exposure is time dependent, Rhodamine B (1 μg/ml) was added to one side of the confined collagen hydrogel in the microdevice and allowed to diffuse across the hydrogel. Time-lapse confocal images showed the gradient evolved along the full width of the central microchamber (Fig 2A and 2B).

We next set out to evaluate the spheroid gradient sensing ability using FDA that was added to one side of the collagen hydrogel within the microdevice and allowed to diffuse across the hydrogel. In live cells, FDA enters through the cell membrane and once inside the cell, it is transformed by the action of esterases into the non-permeable green-fluorescent compound Fluorescein [37]. Thus, cells within the spheroid became fluorescent once they were exposed to the FDA perfused through one of the lateral microchannels. As can be seen (Fig 2C), cells on the surface of the spheroid did indeed show fluorescence, however, the intensity of this fluorescence was not uniform across the spheroid boundary at any stage during the 1 hour experiment (Fig 2D and S2 File). Instead, those cells on the spheroid surface closer to the diffusing chemical front show more fluorescence intensity, suggesting that they are exposed to more FDA. Over the length of the experiment, fluorescence intensity gradually builds up not only in the front of the spheroid, but also to the sides and the back of the spheroid (Fig 2E). Interestingly, whilst there is significant infiltration of the chemical into the spheroid, FDA appears to reach the back of the spheroid before reaching the core. Fluorescence intensity measurement along the spheroid perimeter showed a maximum centered on the region closest to the microchannel perfused with FDA, and decreasing as the distance increases (Fig 2F, red line). This pattern is wholly consistent with a chemical front moving across the microchamber and washing over the spheroid rather than penetrating through it. Since we have already shown the whole of the spheroid contains viable cells (see above) we conclude that this is not due to a necrotic core, but an inability of FDA to penetrate through the spheroid at a rate comparable to the one it diffuses through the medium.

When FDA was flushed manually through the hydrogel, exposure of the spheroid to the chemical was uniform and fluorescence intensity along the spheroid perimeter showed homogeneous distribution (Fig 2F, blue line).

**Fig 3:**
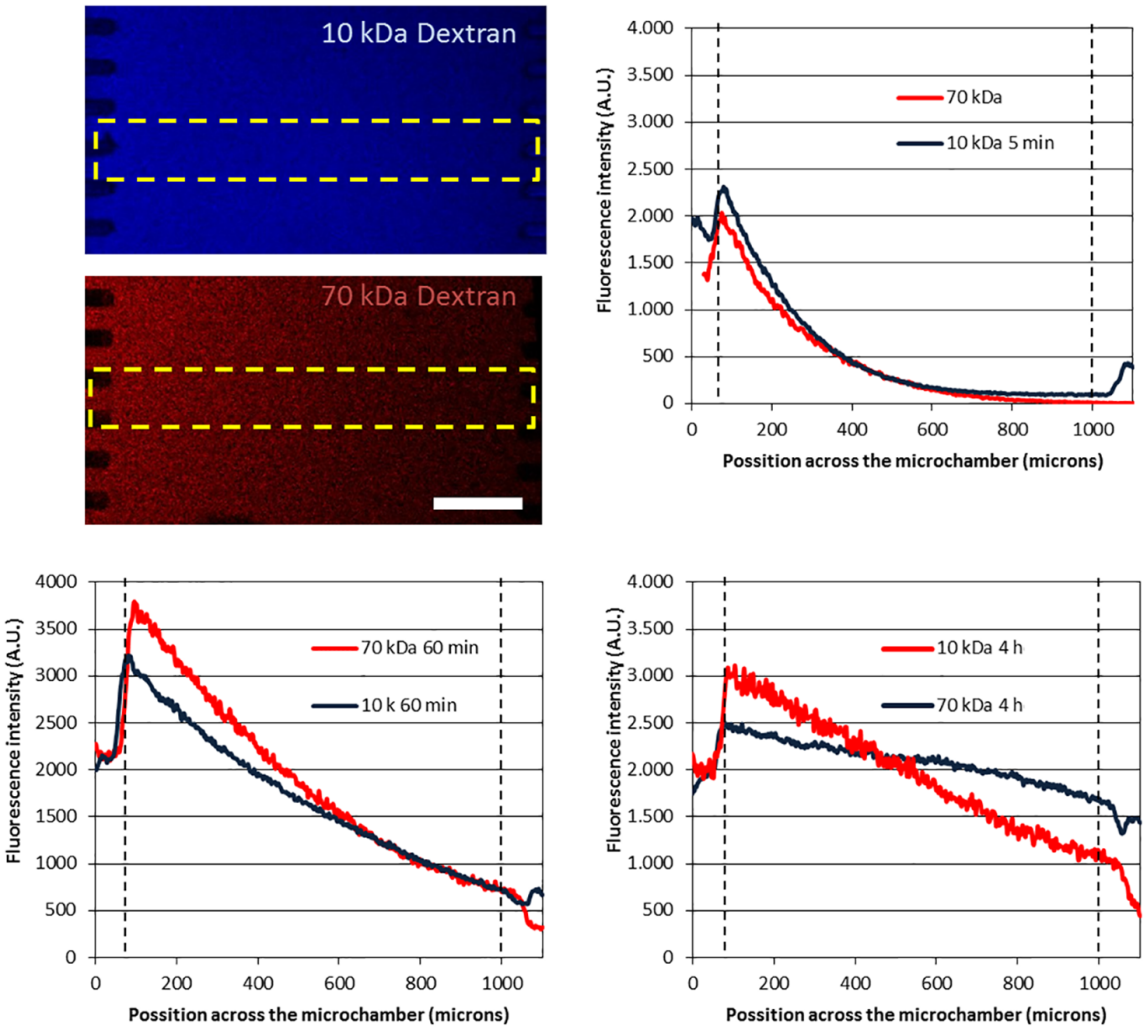
Influence of molecule size on diffusion profile. (A) Cascade blue–10 kDa and TRITC–70 kDa dextran at 10 μM were injected in the left microchannel and confocal images are shown after 4 hours. Fluorescent 10 kDa and 40 kDa diffusion profile is compared at 5 min (B), 60 min (C) and 4 hours (D) post-injection.

Here a FDA/Rhodamine B gradient has been characterized. However in the FBS there is a complex mixture of different growth factors. These different growth factors have different molecular weights, which have a deep impact on the diffusion velocity. To illustrate this, the diffusion profile of fluorescent 10kDa and 70kDa dextrans was recorded using confocal microscopy (Fig 3). The results showed how the larger dextran took a longer time to diffuse through the hydrogel. Many of these FBS growth factors have a molecular weight within this 10 kDa–70 kDa range [38], therefore these results provide a good model for the time-scale of the diffusion profile and show the influence of molecule size on gradient evolution.

### Spheroids migrate against the chemotactic gradient inside the microfluidic device

**Fig 4:**
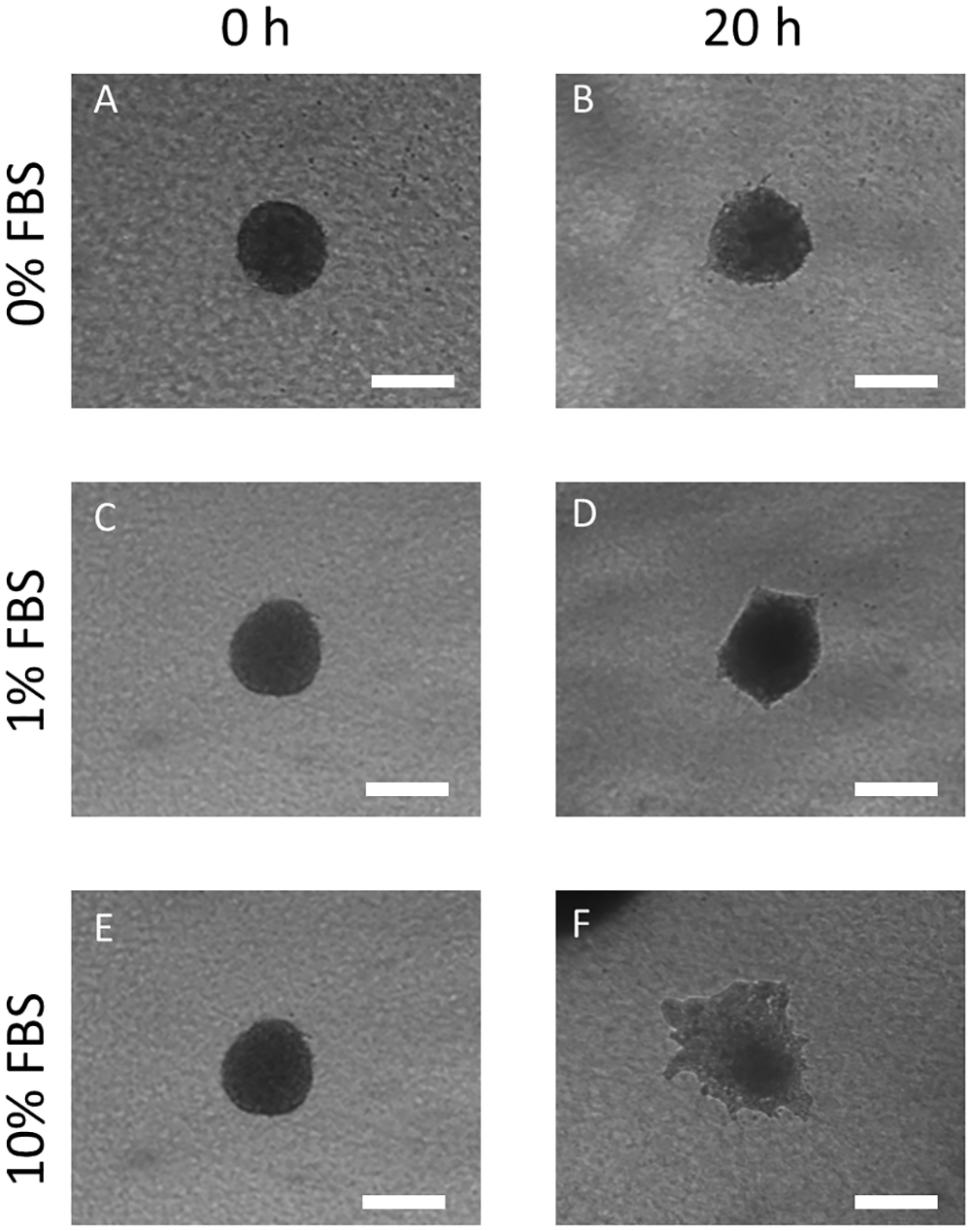
FBS influence on OSC–19 spheroid invasion. OSC–19 spheroids were embedded in collagen hydrogel in a six well plate and growth media supplemented with 0% FBS (A and B), 1% FBS (C and D) and 10% FBS (E and F), was applied over them. Photos were taken at embedding time (0 hours) and after 20 hours. Scale bar is 200 μm.

We next turned our attention to the invasion of OSC–19 spheroids. It is already a well-established principle that cancer cells directionally invade towards a serum enriched environment [17, 18]. To demonstrate that OSC–19 spheroids invade in response to serum, we first carried out a migration experiment outside the confines of the microdevice. OSC -19 Spheroids embedded in collagen were seeded in a 96 well-plate and cultured in complete medium. In this experiment, spheroids experience a uniform (non-gradient) concentration of FBS. We observed that the rate of spheroid invasion depended on the concentration of FBS. Whilst no invasion was observed in 0% FBS, and little invasion in 1% FBS, in 10% FBS there was significant, but non directional and quite random, migration (Fig 4).

**Fig 5:**
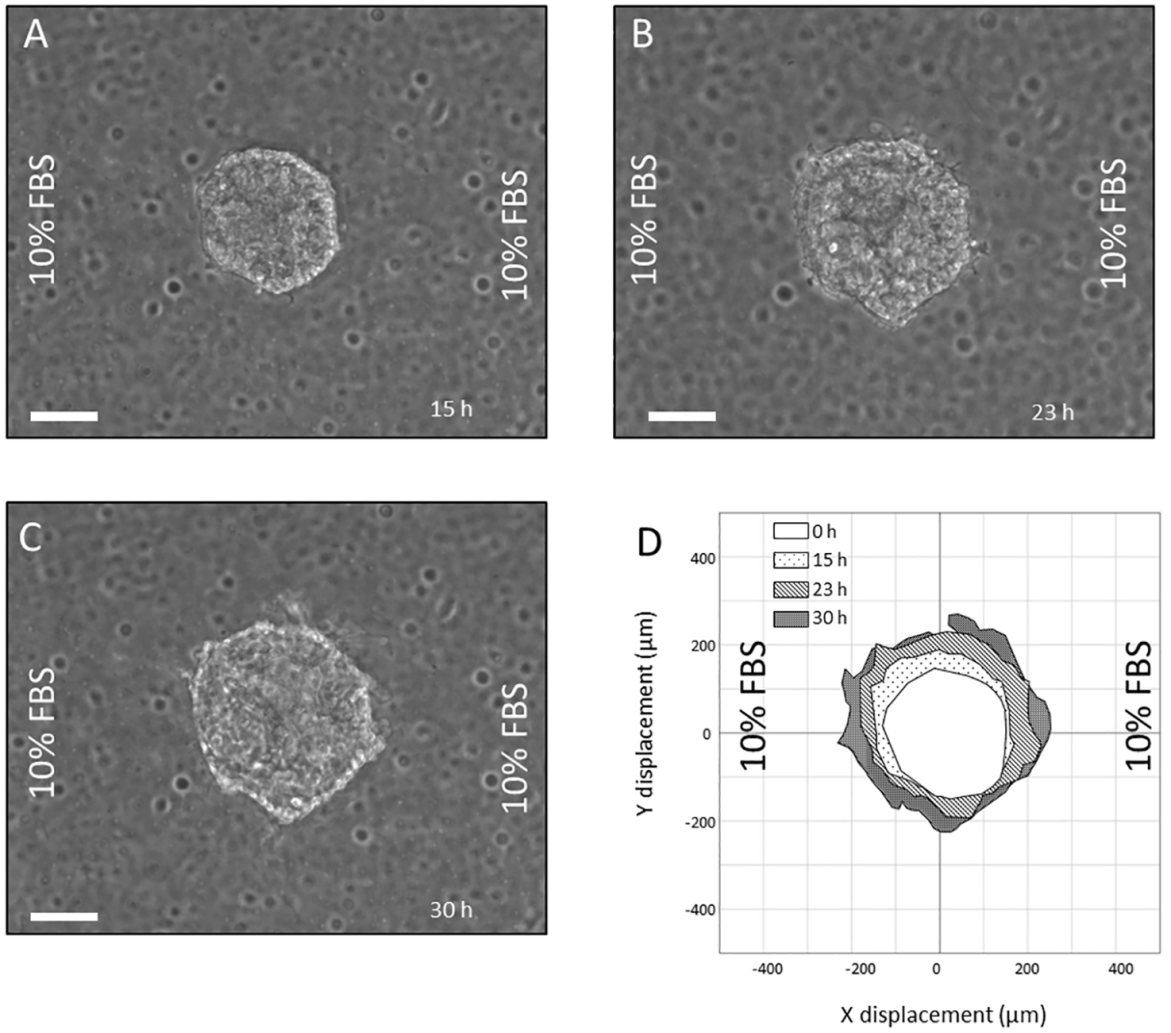
Chemotactic behavior of OSC–19 spheroids under no gradient. OSC–19 spheroids were embedded in collagen hydrogel within the central microchamber. Media supplemented with 10%FBS was perfused through both lateral microchannels. Spheroid invasion is shown after (A) 15 hours, (B) 23 hours, (C) and 30 hours. (D) Area occupied by the spheroid at different times is represented in the graph. Scale bar is 100 μm.

**Fig 6:**
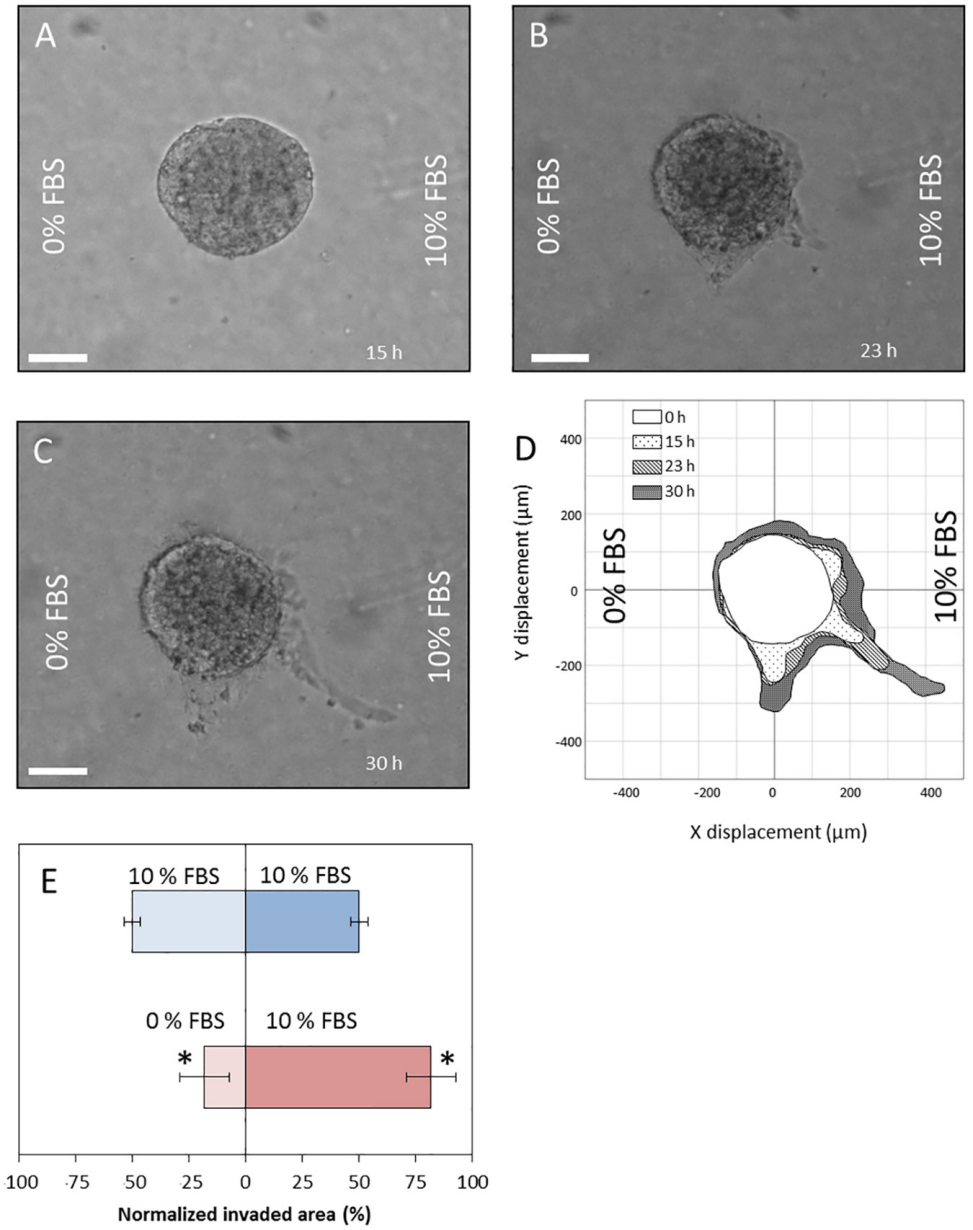
Chemotactic behavior of OSC–19 spheroids under a chemotactic gradient. OSC–19 spheroids were embedded in collagen hydrogel within the central microchamber. Media supplemented with 10%FBS was perfused through right hand lateral microchannel, whereas incomplete media was used in the other. Spheroid invasion is shown after (A) 15 hours, (B) 23 hours, (C) and 30 hours. (D) Area occupied by the spheroid at different times is represented in the graph. (E) Comparison between invaded area under gradient and non-gradient conditions, asterisk denotes difference between both halves is statistically significant (*p*-value < 0.05). Scale bar is 100 μm.

In the microdevice, a FBS gradient was achieved by injecting one of the lateral microchannels with complete media and the other microchannel with incomplete media, establishing a gradient of FBS across the central microchamber. In a control experiment, both lateral microchannels are charged with complete media. For OSC–19 spheroids inside the device, when there was no gradient of serum across the microdevice, i.e. when both channels are charged with the complete growth media, we observed no directional invasion (Fig 5). When the OSC–19 spheroids were subjected to a FBS concentration gradient we observed a clear collective invasion of cells towards that concentration gradient (Fig 6). This migration has also been captured in a time-lapse movie (S1 Movie).

Whilst this invasion was clearly directional with movement towards the FBS gradient, it was not uniform. Indeed, we consistently observed cell offshoots with significantly more invasive response. This is entirely consistent with the proposal that in collective migration, leader cells open up the path of invasion and other cells follow them [39]. It is interesting to note that in view of our earlier observation which showed that chemoattractant waves engulf the spheroid rather than go through it, we believe the invasion is mostly initiated from the outer layers of the spheroid.

Significantly, this invasion is both aggressive (*vide supra*) and coordinated, resulting in cells invading collectively.

### Spheroid invasion versus isolated cell migration

**Fig 7:**
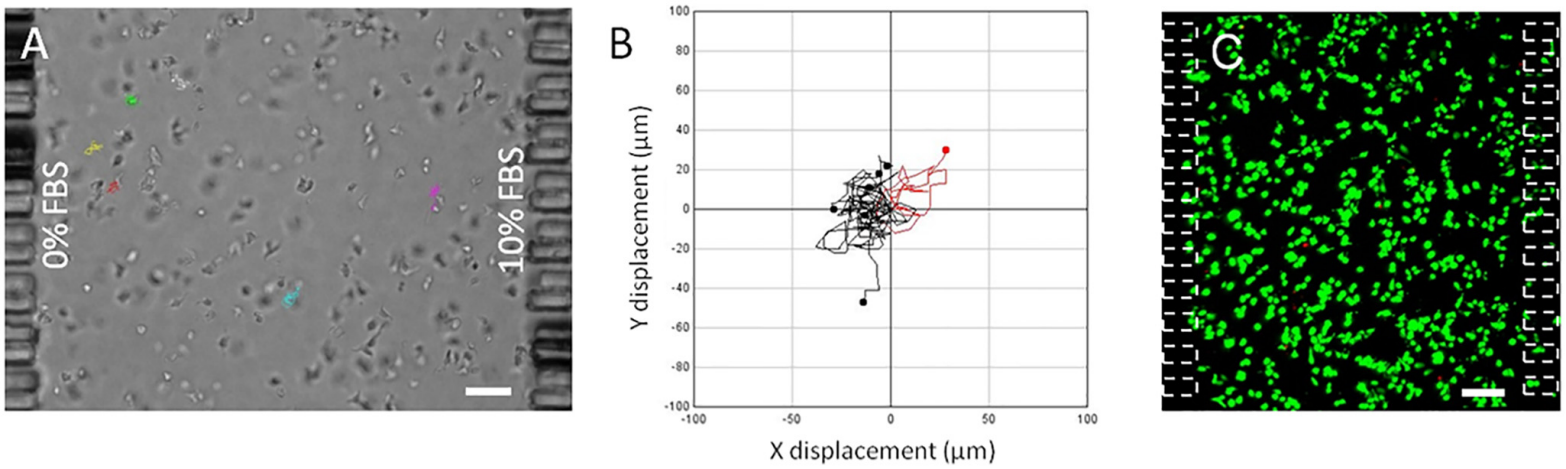
Chemotactic behavior of OSC–19 individual cells. (A) Individual OSC–19 cells were embedded in collagen hydrogel within the central microchamber. Media supplemented with 10%FBS was perfused through one lateral microchannel, whereas basal media was used in the other. Tracks of migrating cells are shown. (B) Individual cell trajectories are plotted, showing those with a net displacement to the right in red, and those displaced to the left in black. (C) Isolated OSC–19 cell viability after 30 hours under gradient conditions, viable cells are shown in green. Scale bar is 100 μm.

Isolated OSC–19 cells where embedded in a collagen hydrogel within the microdevice, and the response to a 10% FBS gradient was evaluated. Isolated cell trajectories were tracked, showing no preferential migratory direction and very low net displacement (Fig 7A and 7B, S2 Movie, at least ten cells were analyzed). Cell viability was measured using FDA/PI at the end of the study, revealing a majority of cells to be viable (Fig 7C). Interestingly, analysis of time-lapse images revealed that spheroid cell velocity and directionality were much higher compared to the isolated cells (Fig 6 and S1 Movie). This observation is in excellent agreement with previous work showing how epithelial cells migrate more intensely when they are allowed to migrate by a collective movement [40, 41]. Given the relatively short time scale of the experiment and the relatively long distance the cell front moves during the experiment, it is unlikely that proliferation plays a major role in the invasion process. Nevertheless, in order to measure any contribution from proliferation to the invasion process, we checked for the expression of Ki–67, a well-established cellular marker of proliferation, in OSC–19 as well as U–87 MG spheroids, by immunofluorescence. For OSC–19 spheroids, the results showed that cells located behind the leading edge of invading cells were negative (S3 File) for Ki–67 although as expected, the population of cells at the invading edges where positive for Ki–67. Whilst the cells on the leading edge are expected to be more proliferative, this result demonstrated that proliferation as well as migration contributes to the invasion process.

### Operation of microdevices can be fine-tuned for other spheroids

**Fig 8:**
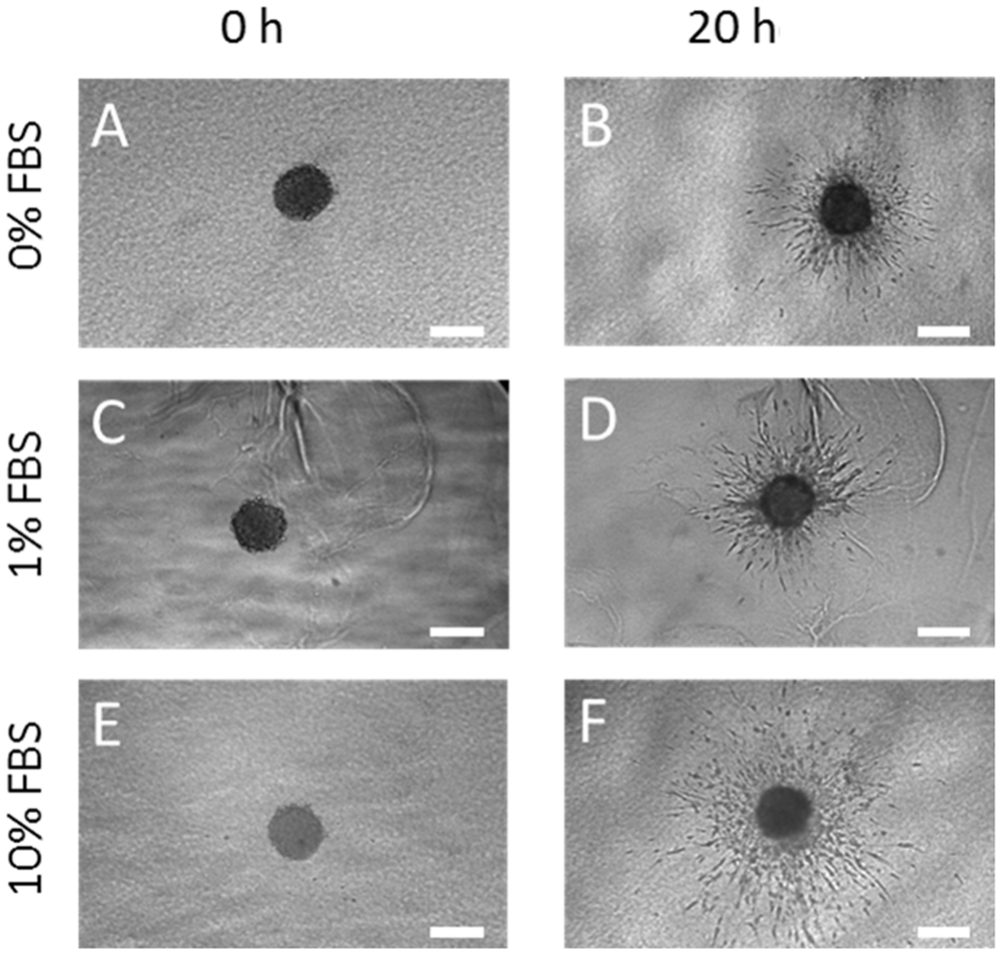
FBS influence on U-87-MG spheroid invasion. U87-MG spheroids were embedded in collagen hydrogel in a six well plate and growth media supplemented with 0%FBS (A and B), 1% FBS (C and D) and 10% FBS (E and F), was applied over them. Photos were taken at embedding time (0 hours) and after 20 hours. Scale bar is 200 μm.

**Fig 9:**
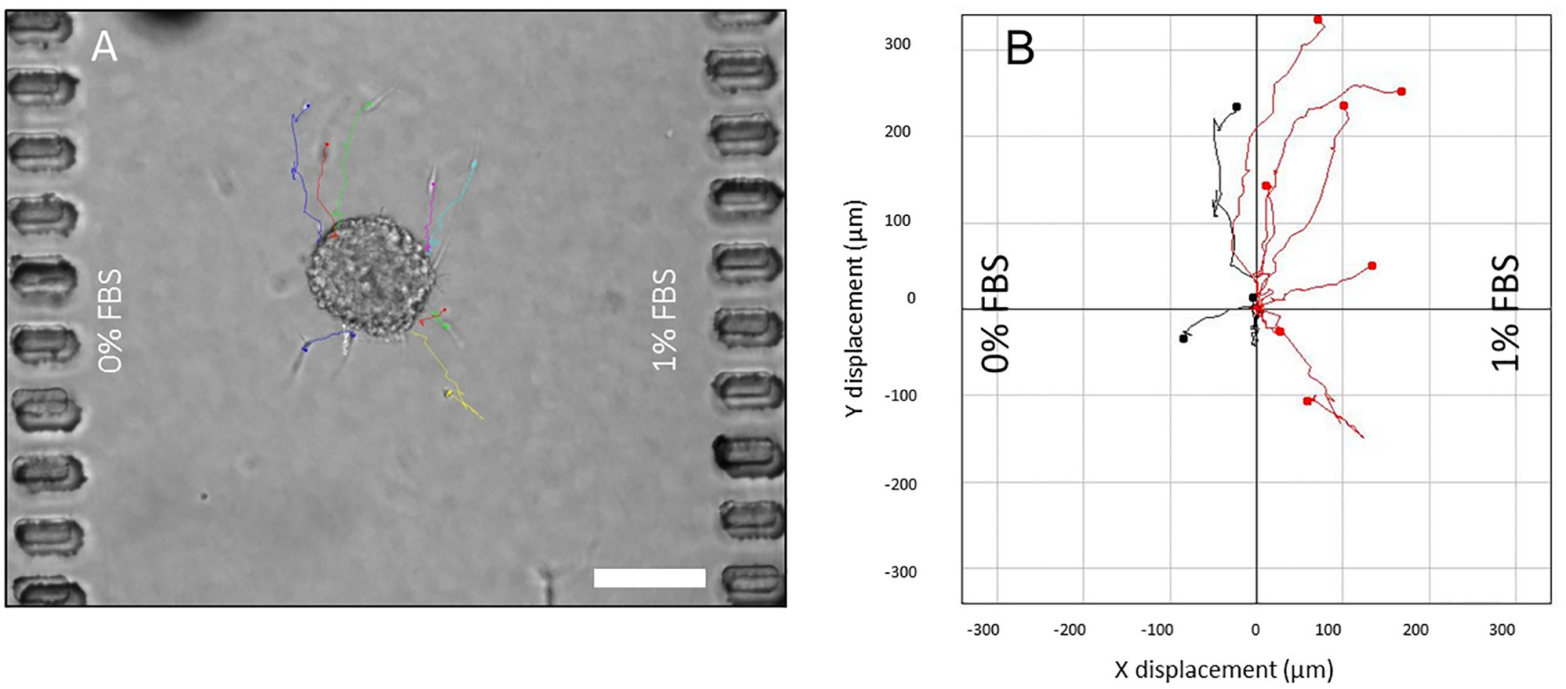
Chemotactic behavior of U-87MG spheroids under a chemotactic gradient. (A) Spheroid was embedded in collagen hydrogel within the central microchamber. Media supplemented with 1%FBS was perfused through one lateral microchannel whereas basal media was used in the other. Brightfield image with overlaid tracks of migrating cells after 12 hours. (B) Individual cell trajectories are plotted, showing those with a net displacement to the right in red, and those displaced to the left in black. Scale bar is 100 μm.

We also investigated migration of U-87MG spheroids. Since these cells are non-endothelial, we expected the pattern of migration to be different. Again, to demonstrate that U87-MG cells migrate in response to serum, we first carried out a migration experiment outside the confines of the microdevice. U87-MG spheroids embedded in collagen were seeded in a 96 well-plate and covered in complete growth medium to experience a uniform (non-gradient) concentration of FBS. As before, we observed that the rate of spheroid migration depended on the concentration of FBS, however in this case, expansion of the spheroid was significantly faster and even at 0% FBS, we observed some expansion of the spheroid (Fig 8). To account for the greater responsiveness of U87-MG cells to FBS, a less steep concentration gradient of FBS was applied across the microchamber by injecting one of the microchannels with growth media containing 1% FBS and the other microchannel with incomplete media (containing 0% FBS). We found that the less steep concentration gradient helped in visualizing migration patterns as there were fewer cells (Fig 9 and S3 Movie). When proliferation was measured, no Ki–67 positive cells were observed (S3 File), demonstrating that U–87 invasion was due only to the migration process.

## Conclusion

Chemotaxis is the principle mechanism by which cells migrate within multicellular organisms. Within this general description, chemotactic processes play different and quite significant roles in many biological processes, and in particular in many developmental and pathophysiological events. Therefore, *in vitro* models that enable the study of chemotaxis have become important tools in biology, particularly those models that provide a closer analogy with physiological systems. In this context, the microdevice we describe herein, and its application to the investigation of the migratory aptitude of multicellular spheroids is a particularly powerful tool.

It is already well established that multicellular spheroids provide a number of advantages over 2D cultures [42–44]. They are considered a more complete model that takes into account cell-cell interactions akin to that observed in real tumors. Our investigations demonstrate further advantages for using microdevices in the study of multicellular spheroids. The control of the chemotactic gradient across the microchamber, coupled to the ability to observe and closely monitor the system over time, makes this a powerful technique for the study of the chemotactic process. In recent years, collective invasion has been proposed as the dominant migration mode during epithelial tumor development [27], and the protocol outlined here enables that process to be observed and analyzed *in vitro*.

Furthermore, microfluidic devices such as the one designed and used here, can clearly have a much wider application. Work is ongoing in our groups to expand the scope of this technique, to study the migratory aptitude towards chemotactic cytokines (chemokines) which are known to play a significant role in cancer cell migration and metastasis; and to study co-cultured multicellular spheroids containing mixed population of cells which better represent the tumor microenvironment.

## Supporting Information

S1 File Design and fabrication of the microdevice. (DOCX)

S2 File Visualization of the chemo-gradient. (DOCX)

S3 File Ki–67 immunofluorescence. (DOCX)

S1 Movie Video showing migration of OSC–19 spheroid under chemotactic gradient. (AVI)

S2 Movie Video showing migration of isolated OSC–19 cells under chemotactic gradient. (AVI)

S3 Movie Video showing migration of U-87-MG spheroid under chemotactic gradient. (AVI)
