# Supplementary material for: Study of the Chemotactic Response of Multicellular Spheroids in a Microfluidic Device
Source: PLoS One. 2015 Oct 7;10(10):e0139515. doi: 10.1371/journal.pone.0139515 (PMC4596573; doi:10.1371/journal.pone.0139515)
Supplement: S1 File — (DOCX) [file pone.0139515.s001.docx]

**Microdevice fabrication**

In order to readily accommodate multicellular spheroids, microdevices were designed with 400 μm diameter inlets and 400μm width and 300μm height microchannels. The central microchamber is 1000µm in width and 300µm high. Microfluidic devices were fabricated using SU-8 photolithography combined with an SU-8 to SU-8 bonding process[[20](#_ENREF_20)]. This fabrication process was inspired by previously reported work describing the fabrication of SU-8 microdevices [[21-23](#_ENREF_21)].

First, a polyimide film (Kapton) was temporarily bonded onto a Pyrex wafer (Figure A). Then, a 60 µm-thick layer of SU-8 was spun onto the Kapton film and soft baked at 65°C for 30 minutes. After the wafer was cooled down to room temperature, a spinning protocol for the deposition of a 20 µm-thick SU-8 layer was performed, followed by an additional soft bake. However, total thickness obtained was 90 µm instead of the expected 80 µm. This can be explained by the higher frictional forces present when layering on top of SU-8 surface compared to Kapton. The wafer was then exposed to a 140 mJ/cm^2^ dose of UV light filtered with a mask that defines the bottom layer of the microdevice. This exposure was followed by a post-bake step, which consisted on heating the wafer up to 65°C for 15 minutes. Three new depositions of SU-8 layers were then performed (60-, 60-, and 20-µm thick), followed by corresponding soft bakes, producing a total height of 150 µm. The wafer was again exposed to UV light (140 mJ/cm^2^) to pattern microchannels and microchambers, then post baked, immersed in SU-8 developer, and finally rinsed in deionised water .

An additional Pyrex wafer was processed with temporary bonding to a Kapton film. The previously described spinning procedures and soft bake processes were applied to this wafer to obtain a 90-µm-thick SU-8 layer. Inlets and outlets were defined by standard photolithography using a 140 mJ/cm^2^ dose of UV light. Then, a 150 µm-thick layer of SU-8 was deposited and patterned with the same mask used to obtain microchannels and microchambers on the first wafer. Both wafers were then aligned and bonded by heating to 90°C for 15 minutes under a pressure of 3 bar. The weak adhesion between SU-8 and Kapton allowed an easy manual release of the devices.


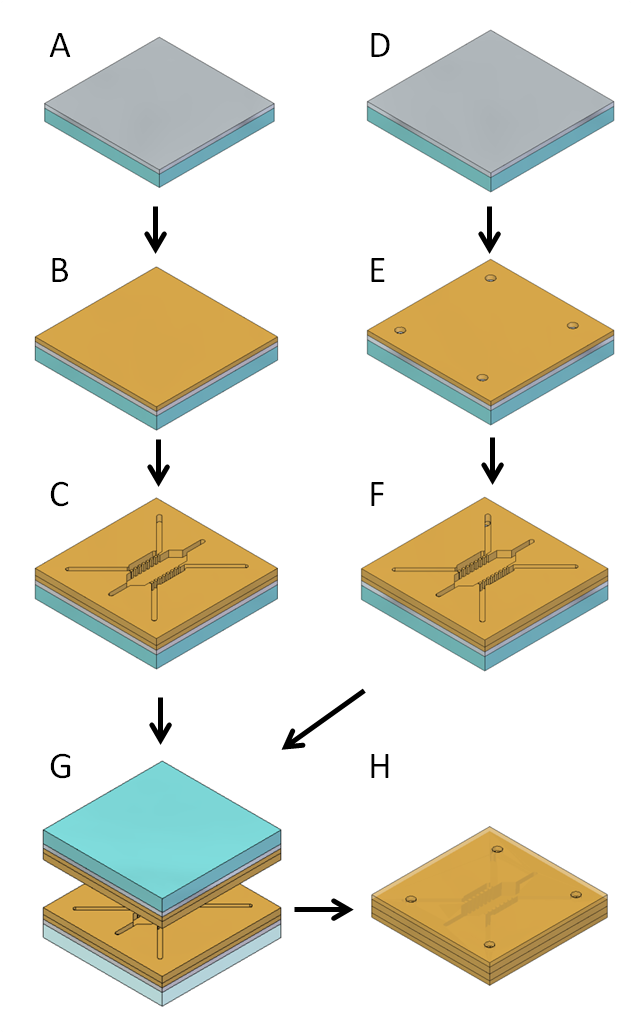


**Figure A.**  Chip fabrication process: A) Kapton film bonding to a Pyrex wafer (grey and blue respectively). B) Processing of a 90 µm-thick SU-8 layer, shown in brown. C) Processing of SU-8 microchamber and microchannels layer. D) Kapton film bonding to a Pyrex wafer. E) Processing of a 90 µm-thick SU-8 layer with the inlets. F) Processing of SU-8 microchamber and microchannels layer. G) SU-8 to SU-8 bonding. H) SU-8 device release.
