# Supplementary material for: Study of the Chemotactic Response of Multicellular Spheroids in a Microfluidic Device
Source: PLoS One. 2015 Oct 7;10(10):e0139515. doi: 10.1371/journal.pone.0139515 (PMC4596573; doi:10.1371/journal.pone.0139515)
Supplement: S2 File — (DOCX) [file pone.0139515.s002.docx]

**Visualization of the combined Rhodamine B and FDA chemo-gradient.**

In order to demonstrate the spheroid enhanced the FDA gradient intensity across the spheroid, whereas Rhodamine B gradient remained unaffected, both compounds were simultaneously injected on the left microchannel (Figure B): After 1 hour confocal images were taken. In "A", overlaid FDA and Rhodamine B fluorescence is shown. In "B" the Rhodamine B fluorescence is shown alone, whereas FDA fluorescence alone is shown in "C". The dark spot near to the spheroid was caused by the presence of an air bubble. Analysis of the fluorescence profile was done on the delimited region to try to avoid the influence of this bubble. In "D" the fluorescence profile of Rhodamine B and FDA can be observed.

Rhodamine B is diffusing passively through the spheroid without creating a significant difference between one spheroid half and the other. On the other hand, FDA showed a significant difference between both halves.

**
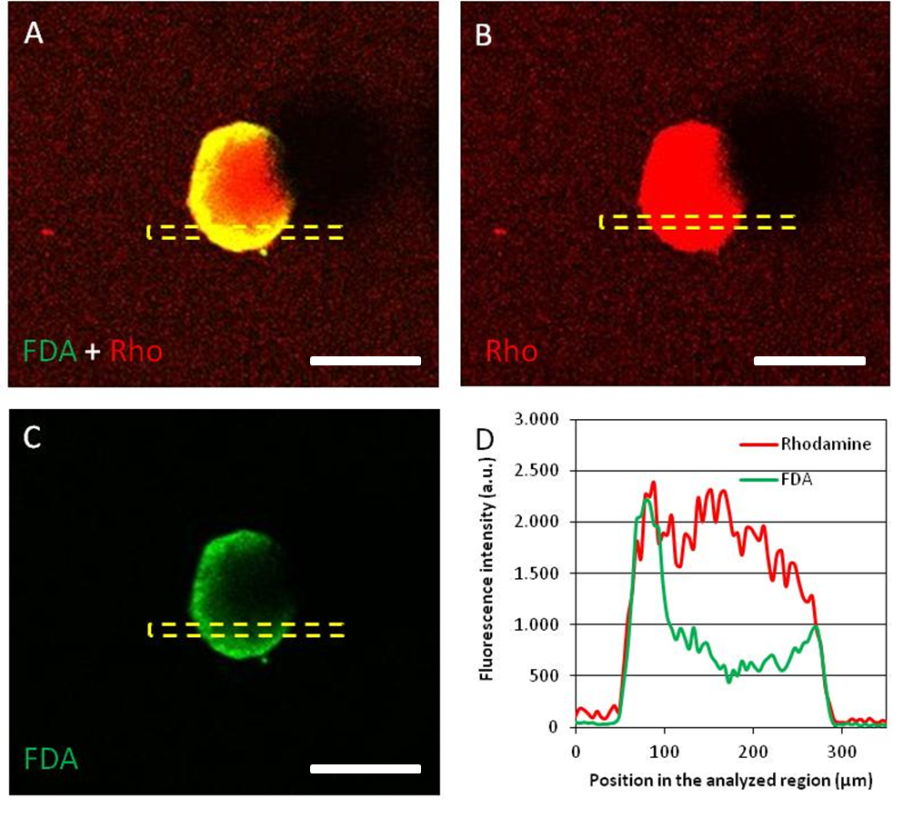
**

**Figure B.**  Combination of Rhodamine B and FDA chemo-gradients. In "A" overlaid Rhodamine B and FDA fluorescence is shown. Rhodamine B fluorescence gradient is shown alone in “B” whereas FDA alone is shown in “C”. In “D” the fluorescent profile along the delimited region is shown. Scale bar is 200 µm.
