# Supplementary material for: Study of the Chemotactic Response of Multicellular Spheroids in a Microfluidic Device
Source: PLoS One. 2015 Oct 7;10(10):e0139515. doi: 10.1371/journal.pone.0139515 (PMC4596573; doi:10.1371/journal.pone.0139515)
Supplement: S3 File — (DOCX) [file pone.0139515.s003.docx]

**Cell proliferation**

Contribution of cell proliferation to the invasion process was evaluated by Ki-67 immunolabelling (Figure C). In OSC-19 spheroids positive cells were observed at the invading edge, whereas cell located behind were negative. On the other hand, in U-87 MG no significant positive cells were observed.


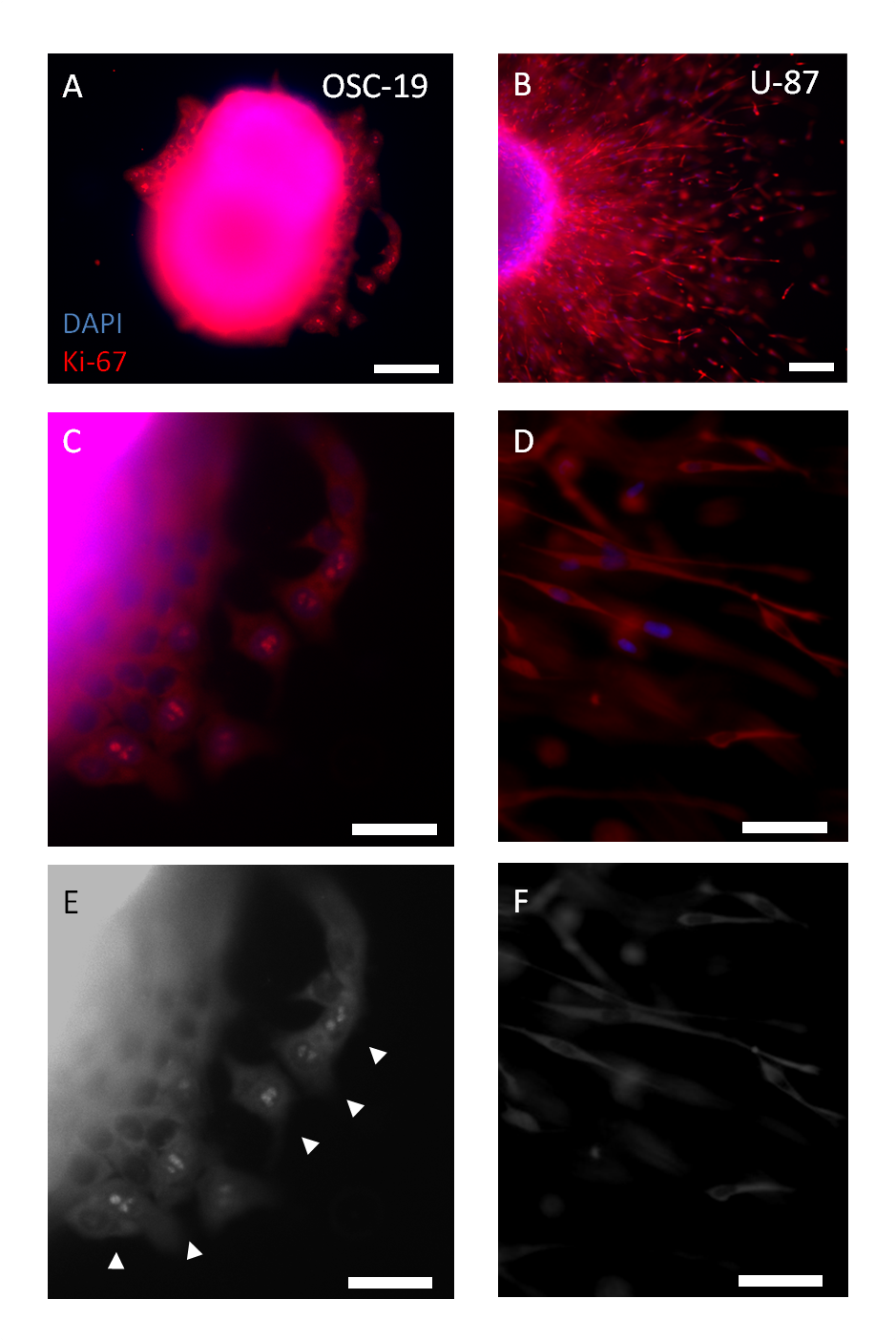


**Figure C.**  Ki-67 immunofluorescence. In "A", "C" and "E" is shown the immunofluorescence for OSC-19 spheroid after 30 hours of invasion. Ki-67 is shown in red and nucleus were labelled with DAPI (Blue). "B", D" and "F" shown the same immunolabelling for U-87 MG spheroids. In "E" and "F" only Ki-67 immunolabelling is shown. Scale bar is 100 μm in "A" and "B" and 20 μm for "C", "D", "E" and "F". Arrows indicate Ki-67 positive cells.
